# The Stability of Metallic MoS_2_ Nanosheets and Their Property Change by Annealing

**DOI:** 10.3390/nano9101366

**Published:** 2019-09-24

**Authors:** Li Li, Jiyang Chen, Keyue Wu, Chunbin Cao, Shiwei Shi, Jingbiao Cui

**Affiliations:** 1 Department of Physics and Materials Science, University of Memphis, Memphis, TN 38152, USA; lli4@memphis.edu (L.L.); jchen10@memphis.edu (J.C.); 2 College of Electrical and Photoelectronic Engineering, West Anhui University, Lu’an 237012, Anhui, China; wukeyue@sina.com; 3 Department of Physics, Anhui Agriculture University, Hefei 230036, Anhui, China; cao_chunbin@outlook.com; 4 School of Physics and Materials Science, Anhui University, Hefei 230039, Anhui, China; 03032@ahu.edu.cn; 5 Department of Physics, University of North Texas, Denton, TX 76203, USA

**Keywords:** MoS_2_ nanosheets, phase transition, annealing, stability, property change

## Abstract

Highly pure 1T MoS_2_ nanosheets were grown at 200 °C by a hydrothermal process. The effects of mild annealing on the structural and physical properties of the MoS_2_ were studied by heating the nanosheets in air and vacuum up to 350 °C. It was found that the annealing leads to an increase in resistivity for the nanosheets by 3 orders of magnitude, the appearance of two absorption bands in the visible range, and a hydrophilic to hydrophobic change in the surface wetting properties. Monitoring of the annealing process by Raman spectroscopy indicates that the material property changes are associated with a 1T to 2H MoS_2_ phase transition, with activation energies of 517 meV in air and 260 meV in vacuum. This study provides another way to control the electrical, optical, and surface properties of MoS_2_ nanosheets for fulfilling the needs of various applications.

## 1. Introduction

The most widely studied 2D material is graphene, a single or few layer graphite sheet, which has potential applications for transparent electrodes, energy storage and catalysis due to its high electrical conductivity, large specific surface area, and other striking characteristics [1,2,3,4]. However, field-effect transistors built from graphene cannot effectively function as electronic switches due to the zero-band gap. To overcome this deficiency of graphene, extensive research has been focused on transition metal dichalcogenides (TMDs), which represent a large family of layered 2D materials. Among them, MoS_2_ has drawn much attention in the last few years due to its unique properties applicable to hydrogen evolution and electronic and optoelectronic devices [5,6].

Depending on the relative alignment of the two atom sheets within a single S-Mo-S sandwiched layer, MoS_2_ shows two different polymorph structures: Thermodynamically stable hexagonal semiconducting 2H MoS_2_ and metastable octahedral metallic 1T MoS_2_ [6]. Both 2H MoS_2_ and 1T MoS_2_ show potential applications in various areas. For example, single layer 2H MoS_2_ has a direct band gap of 1.8 eV [7] and is suitable for a wide range of devices such as Field-Effect Transistors (FETs) [8,9], memory devices [10], solar cells [11,12,13], and phototransistors [14,15,16]. Metallic 1T MoS_2_ nanosheets possess high conductivity and active edge sites, which can be used as a promising alternative catalyst for the replacement of expensive Pt in an efficient and low-cost Hydrogen Evolution Reaction (HER) [17,18,19]. A recent study also shows that 1T MoS_2_ can be used as an electrode material for superfast supercapacitors for energy storage [20].

We have recently realized highly pure 1T MoS_2_ nanosheets by a hydrothermal process, which is promising for energy applications [17]. In this study, the effect of annealing on the 1T MoS_2_ nanosheets was investigated by heating the samples in air and in vacuum up to 350 °C. Following annealing, significant changes in the physical property of the nanosheets were identified by a Hall effect measurement, UV–vis absorption, and water contact angle measurements. A Raman study revealed that the property changes are associated with the phase transition of the nanosheets from 1T to 2H. The underlying mechanism for the phase transition is discussed. The results obtained in this investigation enable one to better understand the materials and to tailor their properties to meet specific applications.

## 2. Experimental Details

The synthesis of 1T MoS_2_ involves a similar process to that used in our previous report [17]. Three chemicals are used for 1T MoS_2_ growth: MoO_3_, thioacetamide, and urea. MoO_3_ powder (CAS number is 1313-27-5) was purchased from the Sigma-Aldrich Company; thioacetamide (CAS number is 62-55-5) was purchased from Acros Organics Company; urea (CAS number is 57-13-6) was purchased from the Fisher Chemical company. Octahedral MoO_3_ was used as the starting material and thioacetamide was chosen as the sulfur source. Moreover, urea, a weak reducing agent, played a key role in the formation of 1T MoS_2_, which can precisely and effectively reduce MoO_3_ to 1T MoS_2_ [17].

Twelve mg of MoO_3_, 14 mg of thioacetamide, and 120 mg of urea were dissolved in 10 mL of deionized water. Then, the solution was placed in an autoclave of volume 25 mL and stirred for 2 h. The autoclave was loaded into an oven heated to a temperature of 200 °C. The temperature of the oven was maintained at 200 °C for 12 h. The reaction was terminated by removing the autoclave from the oven and placing it under running water to cool the solution to room temperature. Black precipitates were first retrieved from the solution and then washed with deionized water followed by centrifugation and sonication. Then, the MoS_2_ sample was stored in deionized water or alcohol for later use. Similar processing and conditions were used to grow 2H MoS_2_ except for the growth temperature which was set to 240 °C.

To study the electrical and surface wetting properties, thin films of MoS_2_ nanosheets were deposited by an airbrush method. Alcohol was chosen as solvent to enable the deposits to dry out quickly. An air blower was also used to blow the substrate for quick removal of solvent. During the deposition, the substrates were mounted onto a hot plate set to a temperature of 50 °C. Each spraying step resulted in a thin layer of deposited material, with the thickness of the films controlled by the number of sprays. The obtained MoS_2_ nanosheets were annealed for 10 min at different temperatures in air and vacuum. The annealing in vacuum was performed in a vacuum chamber with a pressure of 10^−3^ Torr. After each annealing step, material properties including the optical, electrical, and surface properties were studied at room temperature using a Raman microscope, UV–vis absorption spectrometer, Hall Effect measurements, and contact angle measurements. The Raman spectroscopy was measured by using a laser of 532 nm with spot size of about 5 μm and a laser power of 2 mW.

## 3. Results and Discussion

**Figure 1:**
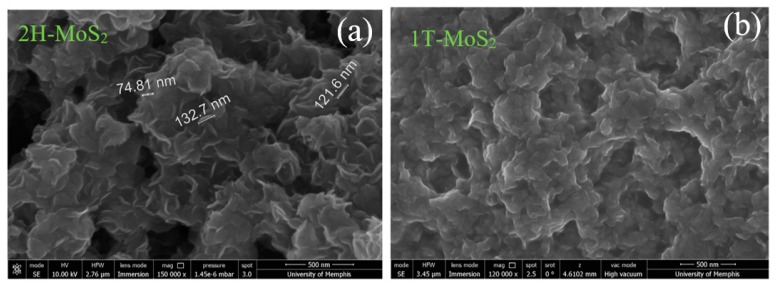
SEM images of 2H MoS_2_ (**a**) and 1T MoS_2_ films (**b**).

The morphologies of as-prepared 2H MoS_2_ and 1T MoS_2_ synthesized by the hydrothermal process are shown in Figure 1. These samples were sonicated for up to 10 min at room temperature to separate out the nanosheets from each other. It can be seen that the 2H MoS_2_ forms clusters even after sonication, which is similar to the morphology of as-grown samples reported previously [17]. The 2H MoS_2_ nanosheets are approximately 100 nm in size, with a thickness of a few nanometers. Without extended sonication, the 1T MoS_2_ nanosheets show a similar morphology as that found for the 2H-MoS_2_ [17]. However, extended sonication separates out the 1T MoS_2_ nanosheets from each other, which results in well dispersed sheets in solution that tend to form a uniform film when dried out on a substrate. This outcome leads to a different microscopic morphology, making it difficult to identify the size and thickness of individual 1T MoS_2_ nanosheets. The difference between 1T and 2H MoS_2_ films can also be observed by the naked eye. The 2H MoS_2_ shows a rough and inhomogeneous surface with a dark color, whereas the 1T MoS_2_ surface is smooth and uniform with a metallic luster.

**Figure 2:**
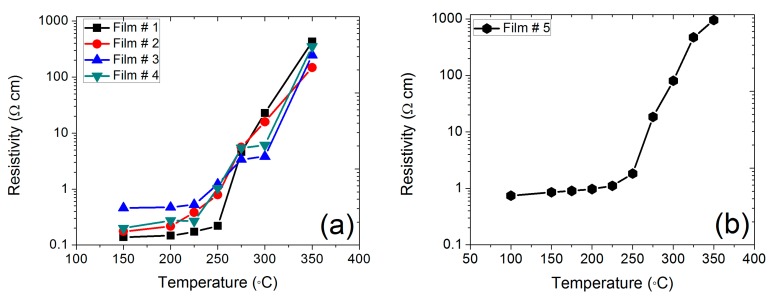
Resistivity of a 1T MoS_2_ thin film annealed in air (**a**) and in vacuum (**b**) at different temperatures.

The annealing of the 1T MoS_2_ nanosheets at different temperatures in vacuum and in air was performed, and their properties were studied after each annealing step. Figure 2 shows the electrical property of the 1T MoS_2_ after annealing as measured by the van der Pauw method. The experiments were repeated for four 1T MoS_2_ nanosheet thin films annealed in air at different temperatures (referred to as sample #1, #2, #3, and #4) with one sample annealed in vacuum (referred to as sample #5). The measured resistivity for the annealed samples is plotted as a function of annealing temperature in Figure 2. All the samples showed a similar trend in resistivity change after annealing in air and vacuum. At temperatures below 225 °C, the resistivity showed only a slight increase with increasing annealing temperature. However, the resistivity increased exponentially at annealing temperatures higher than 225 °C. At an annealing temperature of 350 °C, the resistivity was approximately 3 orders of magnitude higher than that for the non-annealed sample.

**Figure 3:**
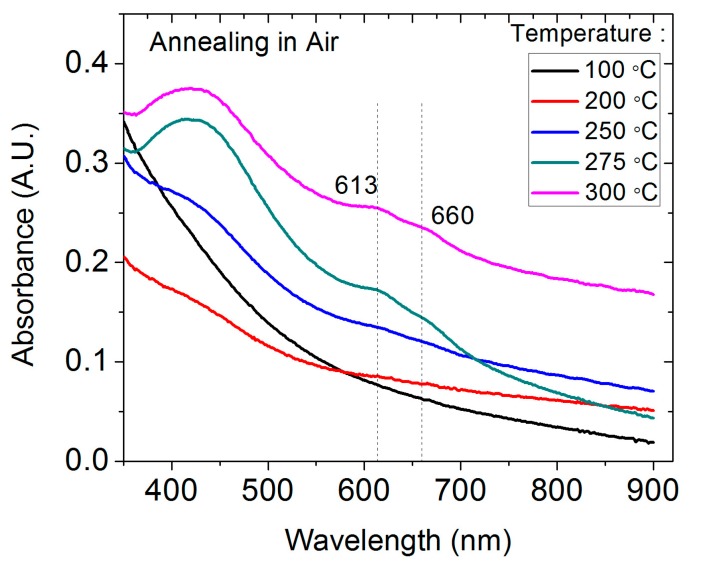
UV–vis absorption spectra for 1T MoS_2_ thin films annealed at different temperatures in air. The vertical dashed lines indicate typical absorption peaks for 2H MoS_2_.

Together with the change in electrical property, the optical absorption of 1T MoS_2_ also showed a significant change after annealing. Figure 3 shows the UV–vis absorption spectra for as-deposited 1T MoS_2_ and samples annealed at different temperatures. The as-prepared 1T MoS_2_ nanosheet film deposited at 100 °C exhibited the typical absorption spectrum expected for metallic MoS_2_, with no salient absorption bands but for a monotonic curve [17]. As the annealing temperature increased, the absorption bands at approximately 450 nm and 600–700 nm gradually developed. Two typical absorption peaks located at 613 and 660 nm were clearly observed after the sample was annealed at 275 °C and 300 °C, which are associated with the 2H MoS_2_. These absorption peaks are associated with the energy splitting due to the valence band spin–orbital coupling in 2H MoS_2_ with large lateral dimensions. These signature absorption bands are intrinsic, which excludes the contribution from the surface absorbates or residue chemical reagents if any. The intensities for these two absorption bands with background subtraction showed no obvious change after annealing at 275 and 300 °C. 

**Figure 4:**
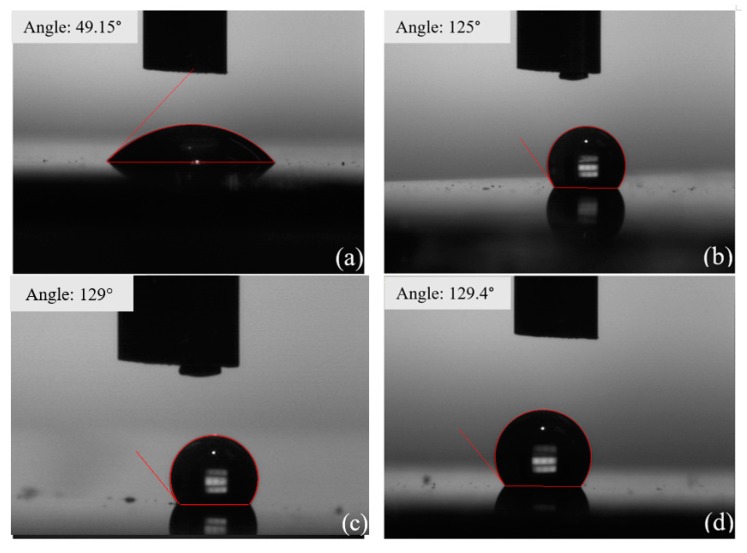
Static contact angle images of a water droplet on as-deposited 1T MoS_2_ (**a**); as-deposited 2H MoS_2_ (**b**); 1T MoS_2_ annealed at 250 °C in vacuum (**c**); and 1T MoS_2_ annealed at 250 °C in air (**d**).

**Figure 5:**
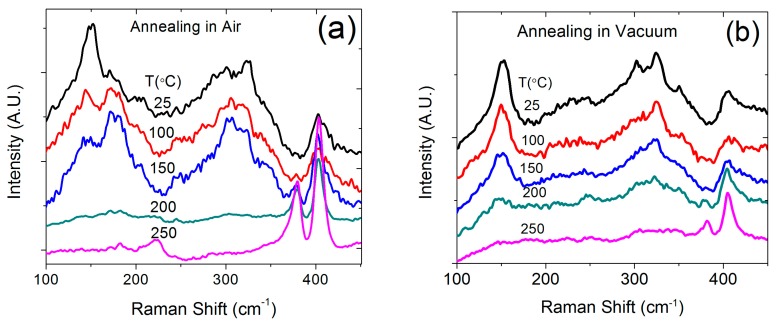

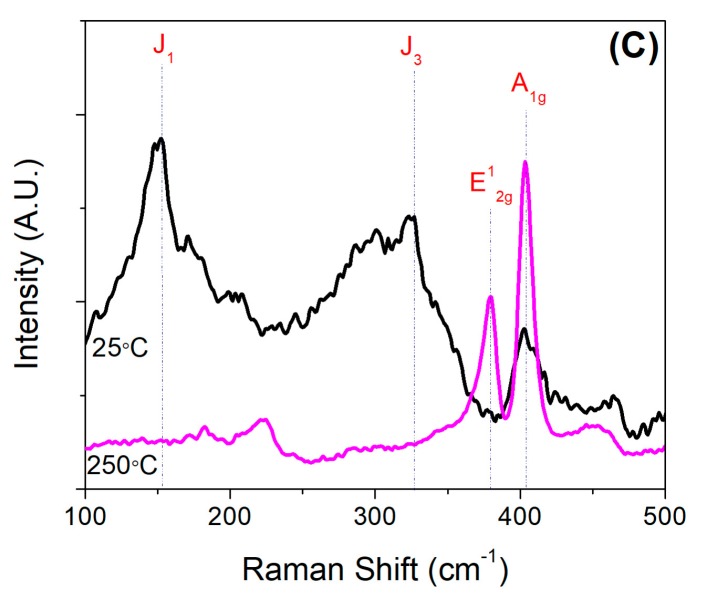
Raman spectra for (**a**) 1T MoS_2_ film annealed at different temperatures in air; (**b**) annealed at different temperatures in vacuum; (**c**) as grown 1T MoS_2_ (1T phase) and post- annealing at 250 °C in air (2H phase).

The surface property change was monitored for the annealed samples. Figure 4 shows the wetting properties for an as-deposited 1T MoS_2_ sample and for samples annealed in vacuum and in air by using water contact angle measurements. For comparison, a freshly prepared semiconducting 2H MoS_2_ film is also included in Figure 4b, which shows a hydrophobic surface with a water contact angle of 125°. The original 1T MoS_2_ film exhibits a hydrophilic surface, with a contact angle of 49.15° (Figure 4a). After annealing at 250 °C in vacuum or in air, the surface becomes hydrophobic, with a similar contact angle of approximately 129° to that measured for the as-grown 2H MoS_2_. Therefore, the 1T MoS_2_ shows a hydrophilic surface, which can turn into a hydrophobic surface by annealing in air or vacuum. To understand the change in property of the 1T MoS_2_ nanosheets due to annealing, Raman spectroscopy was used to monitor the samples during the annealing process, as shown in Figure 5. Raman spectroscopy has been established as a powerful tool in distinguishing the various phases of TMDs through measuring distinct Raman intensity and frequencies for each phase [21]. The Raman spectrum of 2H MoS_2_ had two prominent peaks: an in-plane (E_2g_) mode located around 383 cm^−1^ and an out-of-plane (A_1g_) mode at 407 cm^−1^ [22,23], as magenta traces demonstrate in Figure 5c. Compared to the 2H phase, 1T MoS_2_ has relatively lower in-plane symmetry. The 1T phase shows three Raman active modes that were not present in the trigonal prismatic 2H MoS_2_ polytype [24]. These additional Raman peaks showed up at 150, 226, and 330 cm^−1^ denoted as J_1_, J_2_, and J_3_ [21,22,23,24]. The appearances of these characteristic peaks are attributed to the longitudinal acoustic phonon modes of 1T phase [22]. Therefore, Raman spectroscopy has been used to distinguish the two phases of 2H and 1T MoS_2_. 

In order to reduce the measurement errors in Raman intensity, three spots were selected on each sample by a predefined mark so that the same spots were measured after each annealing step. Our results showed that post-annealed 1T MoS_2_ exhibited Raman features completely different from those of as-grown 1T MoS_2_ at room temperature. One can see from Figure 5a that the as-prepared 1T MoS_2_ (black trace) shows signature Raman peaks at 146 (J_1_) and 330 cm^−1^ (J_3_) for the metallic phase of MoS_2_. The broadening of A_1g_ and E_2g_ modes, as well as suppressed intensity of E_2g_ are also typical to 1T phase. After annealing at 100 °C in air, the intensity of the peak at 146 cm^−1^ was significantly reduced while another peak at 378 cm^−1^ (E_2g_) was observed to emerge. The changes observed in Raman spectra upon annealing imply that samples were 1T-2H hybridized MoS_2_ at this stage. Following a further increase in the annealing temperature, the Raman peak due to the metallic phase gradually disappeared and the peaks at 378 (E_2g_) and 403 cm^−1^ (A_1g_) due to 2H MoS_2_ became stronger. Samples post-annealing treatment at 250 °C in air (magenta trace in Figure 5c) showed clear A_1g_ and E_2g_ vibrational modes corresponding to the 2H phase. The Raman spectra for the samples annealed in vacuum showed a similar trend as that found for the samples annealed in air. However, a slight difference is observed in terms of the peak intensity change, i.e., it changes faster in air than in vacuum. After annealing at 250 °C, the 1T MoS_2_ peak in the Raman spectra became difficult to observe while the 2H MoS_2_ peaks became significant. These experimental data clearly indicate that annealing at mild temperatures induces a transition in the MoS_2_ from the 1T to 2H phase, which explains the related property changes.

As shown by Raman measurements, the phase transition occurred strongly at approximately 150 °C in air and 200 °C in vacuum. However, a change in the electrical resistivity was not obvious at this temperature. We believe this discrepancy is caused by the significant difference in electrical contribution from the two phases. The initial phase transition from metal to semiconductor does not have a significant impact on the resistivity of the film because the electrical transport is still dominated by the metallic phase. Only when a significant amount of the metallic phase becomes semiconducting will the resistivity show an obvious change. In contrast, the change in Raman signal directly reflects the amount of phase transition.

The phase transition occurs due to thermal energy at high temperature during annealing. In terms of kinetic theory, the rate of reaction (phase change) is proportional to the probability for reaching another state, i.e., the reaction rate constant can be described using the following Arrhenius equation,

(1) $$k\left(T\right)=Ae^{-E_{a}/K_{B}T}$$

where *k(T)* is the reaction rate constant in units of s^−1^ at a given temperature *T*. *E_a_* is the activation energy; *A* is the frequency factor, which varies with different reaction conditions; and *K_B_* is the Boltzmann constant of 8.617 × 10^−5^ eV/K.

The Raman spectral change can be used to calculate the activation energy for the phase transition by considering the intensity of the 146 cm^−1^ Raman peak as an indicator for the amount of metallic phase in the material. In this case, the reaction rate constant *k* is reflected by the rate of change in the Raman intensity. Assuming that the phase transition is a first-order thermally activated process at a constant temperature *T*, the intensity of the peak at 146 cm^−1^ can be described as a function of annealing time *t* by I(t) = I(0) $e^{-k\left(T\right)t}$ [25,26]. Note that the rate constant *k* is a function of temperature and constant only at a given temperature. 

**Figure 6:**
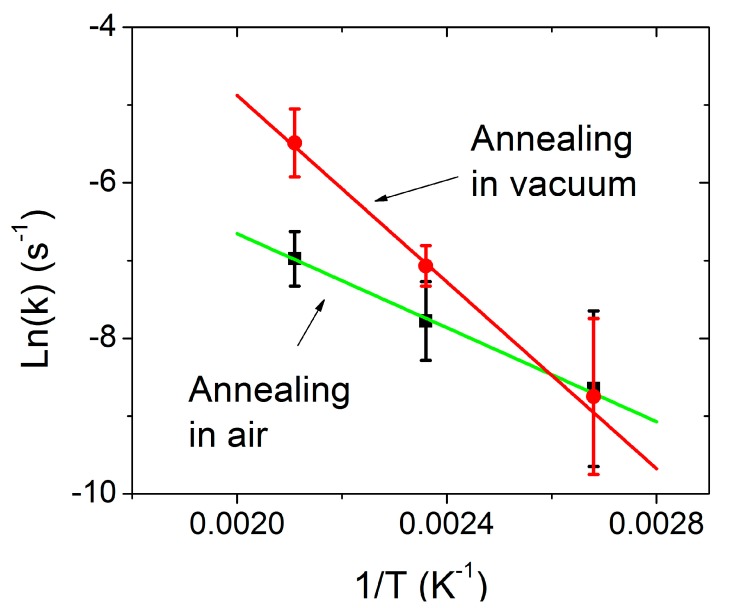
Arrhenius plot of the rate constant *k* for 1T MoS_2_ samples annealed in vacuum and in air. Straight lines are a linear fit to the experimental data.

By plotting *ln*(*k*) as a function of 1/T, the activation energy can be calculated from the slope, as shown in Figure 6. The activation energies for the metallic to semiconducting MoS_2_ phase transition are determined to be 517 meV in vacuum and 260 meV in air. The activation energies obtained fall in the range of the value of 400 ± 60 meV reported by Guo et al. for exfoliated samples [27]. Though the calculated activation energy may have significant errors up to 139 meV based on the error bars shown in Figure 6, it is obvious that the phase transition depends on the annealing atmosphere. The different activation energies in air and in vacuum may be related to the presence of oxygen during annealing in air, which assists in the phase transitions. 

As reported in previous studies, the Raman scattering at 146 cm^−1^ is the signature of metallic phase of MoS_2_ while the double peaks at 378 and 403 cm^−1^ are the fingerprints of the semiconducting phase [21,22,23,24]. It provides one of the easy and nondestructive ways to directly monitor the transition process of MoS_2_. Several groups have confirmed the formation of 1T MoS_2_ from the emergence of new Raman shifts associated with the phonon modes of 1T MoS_2_ [21,24,28,29,30]. Therefore, the Raman data clearly provide the key evidence of phase transition in MoS_2_ nanosheets. Although other approaches such as x-ray diffraction could also be used to study the phase transition in layered structures, no obvious differences were observed between the metallic and semiconductor phases. 

**Figure 7:**
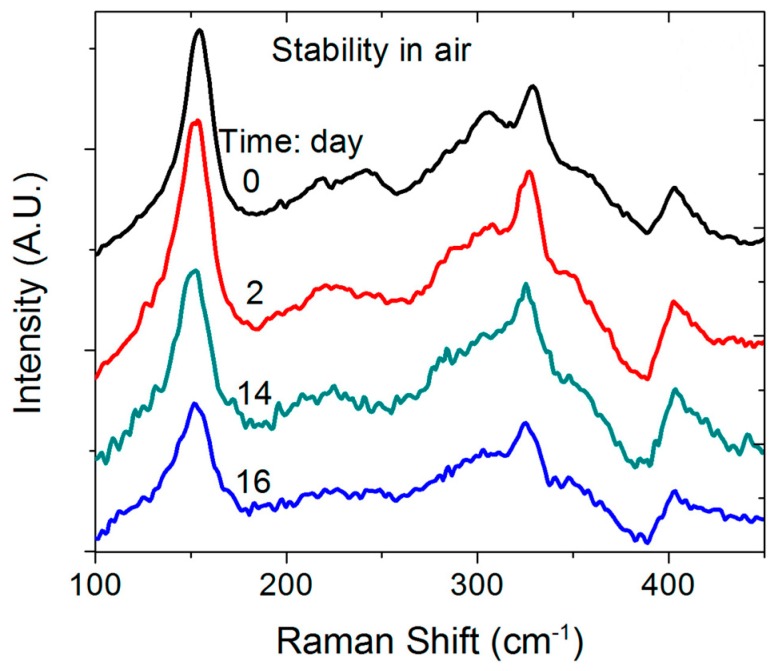
Raman spectra for 1T MoS_2_ stored in air for different times.

The activation energy obtained in this study was well above the thermal energy of 25 meV at room temperature, which suggests that at room temperature 1T MoS_2_ should be relatively stable even though it is supposed to be metastable. Figure 7 shows Raman spectra measured for 1T MoS_2_ nanosheets stored in air for up to 16 days. One can see that there is a slight reduction in the intensity of the Raman peak at 146 cm^−1^ obtained for longer storage times, with the 2H MoS_2_ peak at 378 cm^−1^ not obviously developed after 16 days, confirming that the 1T MoS_2_ is relatively stable at room temperature.

The phase transition in MoS_2_ is a process which largely depends on the electron donor as well as the thermal environment [5,31]. In other words, the actual dynamical process of the transition involves intra- and inter-layer atomic plane gliding, which is caused by atom displacement and extra thermal energy. The 2H MoS_2_ has a hexagonal lattice with a threefold symmetry and an atomic stacking sequence (S-Mo-S’) of ABA [5]. Each Mo atom in the 2H phase lies in a center prismatically coordinated by six surrounding S atoms, with the S atoms in the upper layer lying directly above those of the lower layer. In contrast, the Mo atom in the 1T phase is octahedrally coordinated to six neighboring S atoms, with an atomic stacking sequence of S-Mo-S’ as ABC, where the bottom S’ plane occupies the hollow center (HC) of the top S lattice. Theoretical calculations showed an energy of 0.18 eV/fu (fu = formulae unit) for interlayer S/S gliding and 1.8 eV/fu for intralayer Mo/S gliding within MoS_2_ crystals, clearly indicating that interlayer gliding is the favored mechanism in MoS_2_ (trigonal structure) [6].

The most extensively discussed method for realizing this phase transition is through chemical exfoliation [5,27,32,33,34]. It has been reported that during an electrochemical process, charges mainly accumulate on the S atoms and are depleted in the area between the S and Mo atoms. The extra electrons weaken the Mo−S bonds and promote atomic gliding towards the less atomically concentrated area [5,34]. Meanwhile, the extra electrons transferred into the nonbonding d-orbitals of 2H MoS_2_ make it isoelectronic with group 7 TMDs with metallic character and decrease the relative energy of 1T MoS_2_. Therefore, ion intercalation can destabilize the 2H phase and reduce the barrier for the 2H to 1T phase transition to some extent [34]. This explains why 1T MoS_2_ nanosheets are obtained after intercalation. 

The transition of chemical exfoliated 1T MoS_2_ to 2H MoS_2_ by annealing was recently studied [27,32]. Several mechanisms for explaining the phase transition and stability of 1T MoS_2_ have been proposed, including a doping effect, ion intercalation, and surface functionalization [35,36]. The annealing induced phase transition reported in this study is associated with the destabilization of 1T MoS_2_ at moderate temperatures (150–200 °C). The basic process involves sulfur atomic plane gliding to hollow center sites due to thermal energy [5], but no detailed mechanism of the restoration process is revealed so far. It is likely that the 1T to 2H phase transition is driven by the extra thermal energy obtained by 1T MoS_2_ at high temperature, which leads to sulfur atom gliding towards the hexagonal structure of the 2H phase. This outcome is consistent with the observations by Eda et al. [32] and Guo et al. [27], indicating that the Raman features due to 2H MoS_2_ appear after annealing. 

There are several possibilities to stabilize 1T MoS_2_ despite pure MoS_2_ being metastable. By using scanning transmission electron microscopy, Lin and colleagues directly observed the phase transition process of single-layered MoS_2_ that was controllable by an electron beam [5]. Similarly, Enyashin et al. observed that the stabilization of 1T MoS_2_ can be realized by re-doping, as was shown by high resolution transmission electron microscopy and density functional calculations [35]. In a recent calculation, Tang and Jiang found that 1T MoS_2_ has a strong affinity for functional groups, which is closely related to its metallicity and partially filled Mo 4d states [36]. Interestingly, they also found that 1T MoS_2_ is metastable when un-functionalized but becomes a stable phase following a crossover coverage of functionalization. In our hydrothermal growth of 1T MoS_2_ nanosheets in solution, the surface of the nanosheets is likely functionalized by other functional groups, which help stabilize the 1T MoS_2_ nanosheet materials. Further study is needed to identify the details of the functional groups including comparison with theoretical calculations to better understand the stability of the 1T MoS_2_ nanosheets observed in this study.

## 4. Conclusions

In summary, the effect of annealing on the properties of 1T MoS_2_ nanosheets grown by a hydrothermal process was investigated. Annealing 1T MoS_2_ at mild temperatures in air or in vacuum causes significant changes in the electrical transport, optical absorption, and surface wetting properties. After annealing, the resistivity of 1T MoS_2_ nanosheets increases from 10^−1^ to 10^3^ Ohms per square, the hydrophilic surface becomes hydrophobic, and typical semiconducting bands appear in the optical absorption spectrum at 613 and 660 nm. Raman spectroscopy measurements indicate that these property changes are associated with a temperature-induced phase transition from 1T to 2H MoS_2_, with an activation energy of 517 in vacuum and 260 meV in air. The properties of the annealed 1T MoS_2_ are controllable by annealing, which may be potentially useful for device fabrication.
